# Daily phytate intake increases adiponectin levels among patients with diabetes type 2: a randomized crossover trial

**DOI:** 10.1038/s41387-023-00231-9

**Published:** 2023-02-28

**Authors:** Pilar Sanchis, Paula Calvo, Antelm Pujol, Rosmeri Rivera, Francisco Berga, Regina Fortuny, Antonia Costa-Bauza, Felix Grases, Luis Masmiquel

**Affiliations:** 1 Laboratory of Renal Lithiasis Research, Department of Chemistry, University of Balearic Islands, Institute of Health Sciences Research [IUNICS- IdISBa], 07122 Palma of Mallorca, Spain; 2 CIBER Fisiopatología de la Obesidad y Nutrición (CIBERObn), Instituto de Salud Carlos III, Madrid, Spain; 3 Vascular and Metabolic Diseases Research Group, Endocrinology Department, Son Llàtzer University Hospital, Institute of Health Sciences Research [IdISBa], 07198 Palma of Mallorca, Spain

**Keywords:** Type 2 diabetes, Diabetes complications

## Abstract

**Aim:**

Adiponectin, a major adipokine secreted by adipose tissue, has been shown to improve insulin sensitivity. Myo-inositol hexaphosphate (phytate; InsP6) is a natural compound that is abundant in cereals, legumes, and nuts that has demonstrated to have different beneficial properties in patients with diabetes type 2.

**Methods:**

We performed a randomized crossover trial to investigate the impact of daily consumption of InsP6 on serum levels of adiponectin, TNF-alpha, IL-6, and IL-1beta in patients with type 2 diabetes mellitus (T2DM; *n* = 39). Thus, we measure serum levels of these inflammatory markers, classic vascular risk factors, and urinary InsP6 at baseline and at the end of the intervention period.

**Results:**

Patients who consumed InsP6 supplements for 3 months had higher levels of adiponectin and lower HbA1c than those who did not consume InsP6. No differences were found in TNF-alpha, IL-6, and IL-1beta.

**Conclusion:**

This is the first report to show that consumption of InsP6 increases plasma adiponectin concentration in patients with T2DM. Consequently, our findings indicate that following a phytate-rich diet has beneficial effects on adiponectin and HbA1c concentrations and it could help to prevent or minimize diabetic-related complications.

## Introduction

Adiponectin is one of the most prevalent and extensively investigated adipokines and it has been described as an important protective factor against the negative metabolic and cardiovascular effects of diabetes and obesity [1].

Adiponectin, a prominent adipokine released by adipose tissue [2], has received a lot of interest because of its anti-inflammatory and insulin-sensitizing effects, as well as its involvement in glucose metabolism [3]. Adiponectin, tumor necrosis factor-alpha (TNF-alpha), interleukin (IL-6), leptin, and many other chemicals (known as adipokines) are secreted by adipose tissue [4, 5].

Adiponectin enhances insulin sensitivity in both animal and human studies [6, 7]. As a result, decreased serum adiponectin concentrations may be related with a greater prevalence of type 2 diabetes mellitus (T2DM). Animal studies demonstrated an improvement in insulin resistance following adiponectin infusion [8]. Furthermore, calorie restriction increased its beneficial effects on glucose and lipid metabolism [9]. Many investigations have been conducted to determine whether modifiable variables can enhance the physiological levels of adiponectin, with dietary and lifestyle habits being some of the most important changeable factors that can improve blood adiponectin concentrations [10]. In terms of dietary components, high fiber content, fish-rich diet, legume-rich diet, low-glycemic index diet, and low-calorie diet are some of the dietary patterns that have been shown to enhance adiponectin levels [11, 12].

In addition, interventional and epidemiological clinical studies have shown a beneficial relationship between legume consumption and adiponectin levels in type 2 diabetes patients [13]. Legumes are regarded as a nutritious food with several health benefits. Phytate is abundant in legumes and nuts. Phytate (myo-inositol hexaphosphate InsP6) is a naturally occurring chemical found in considerable quantities in whole grains. As a result, persons on a balanced diet usually consume 1–2 g per day of phytate. InsP6 and its phosphorylated derivatives (InsP5, InsP4, InsP3, and InsP2) are present in all mammalian tissues and fluids [14, 15], and their levels vary depending on whether they are taken orally [14–16] or topically [16–18]. In this regard, without the presence of phytates in the diet, the urine content of inositol phosphate (InsPs) drops to undetectable levels after 22 days [14, 15]. Phytate has several therapeutic qualities in diabetes and pre-diabetes, according to many research. InsP6 suppresses insulin secretion [19], which may indicate insulin sensitivity. Furthermore, it has been linked to a reduction in serum lipids [20]. According to Dilworth et al., InsP6 supplements that have been demonstrated to be useful in the management of diabetes have been shown to reduce blood glucose [21]. According to the authors of a research that looked at the effects of phytic acid supplementation on streptozotocin-induced diabetic rats, phytic acid supplementation may be advantageous in the treatment of diabetes mellitus since it has been proven to elevate specific adipokine levels [22]. So far, the effect of phytate supplementation on adiponectin levels has not been studied yet.

We postulated that inositol and InsP6 supplementation would raise blood adiponectin levels. The aim of this study was to see if daily phytate ingestion may increase serum adiponectin levels for up to 3 months in type 2 diabetic patients in a randomized crossover trial.

## Materials and methods

### Subjects and study design

**Fig. 1:**
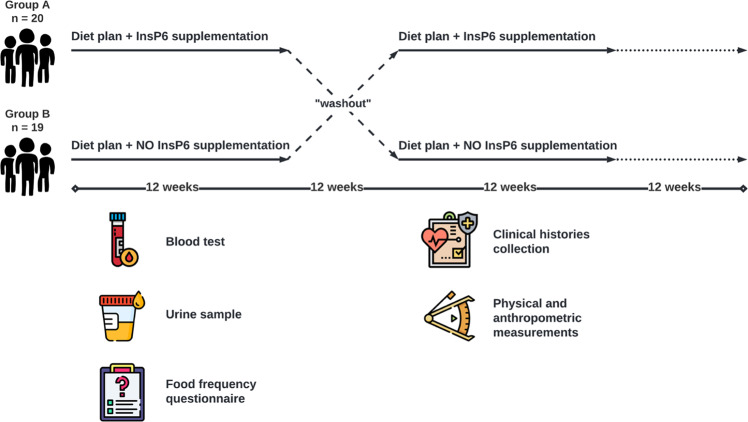
Subjects were randomly assigned to the InsP6-diet group or to the non-InsP6-diet group for 12 weeks. After 12 weeks, each group was given a 12-week washout period, and then switched to the alternate diet for 12 weeks. All patients were then followed for an additional 12 weeks. Patients received an InsP6 diet (diet plan with InsP6 supplementation) or a non-InsP6 diet (the same diet plan without InsP6 supplementation). Blood test, urine samples, food frequency questionnaire, clinical histories, and physical and anthropometric measurements were prospectively collected during the trial.

A single-center, randomized, crossover, open-label study on 39 patients living with Type 2 Diabetes Mellitus (T2DM) was carried out by the Endocrinology Department of Hospital Son Llàtzer (Balearic Islands, Spain). Patients were randomly allocated to one of two cohorts: one was put on a diet plan with InsP6 supplementation for 12 weeks, while the other was put on an identical diet plan without InsP6 treatment. Following that, each group was given a 12-week washout period before switching to the other procedure for another 12-week period. Finally, all patients were followed for another 12 weeks (Fig. 1).

The InsP6 supplementation group consumed one pill of 380 mg calcium-magnesium InsP6 (Broken Laboratorios, SALVAT S.A.) with meals three times each day. Both groups followed a five-meal-a-day diet that comprised fruits, vegetables, fish, shellfish, meat, eggs, olive oil, and low-fat dairy items. This diet was created in Spain to give the appropriate quantities of calories, macronutrients, fiber, minerals, and vitamins for patients with T2DM. Patients were especially counseled not to consume foods high in InsP6 (cereals, legumes, nuts), but instead to substitute white bread, white rice (not basmati), pasta, white flour, potatoes, and non-whole wheat semolina. A trained dietician provided oral and written information on their food plans to all patients, as well as assistance and monthly check-ins. Patients were strongly asked to strictly adhere to the offered instructions and to report any changes in their food consumption (i.e., any deviations from the allotted diet). The dietician assessed supplementation (pills were counted to ensure a minimum of 80% supplement use) and dietary compliance at each appointment.

Inclusion criteria included: (i) being 18 years or older; (ii) having stable glycemic control with no medication modifications in the past 3 months; and (iv) having a low food intake of InsP6 (as determined by dietary questionnaires). During the experiment, participants were told not to alter their anti-hyperglycemic medicine (dose or/and drug) or daily insulin demand by more than 10% unless they encountered a moderate glycemic excursion (fasting capillary glucose levels equal to or more than 250 mg/mL) for 2 consecutive days or category III hypoglycemia.

With 80% power and a 95% confidence interval, the minimal number of patients required to detect a 20% difference in adiponectin levels at the conclusion of the trial was 30 patients per group.

An impartial researcher with no clinical participation in the experiment produced the randomization sequence using Excel software (Microsoft Office 2010) and stratified it with a 1:1 allocation using a random block of four.

An impartial researcher oversaw the allocation consignment recruiting process, enrolling and evaluating individuals in a sequential sequence. There was no blinding after the randomization procedure for either the patients who participated in the trial or the researchers who delivered the various regimens. There was, however, blinding for researchers analyzing results until the end of the trial.

### Variable outcomes

Adiponectin levels were the primary outcome measure of this study. Glycated hemoglobin (HbAc1), fasting glucose, insulin, homeostatic model assessment (HOMA) of insulin resistance, lipids, blood pressure, and weight were secondary outcomes.

Patients’ clinical history might be accessed via electronic medical records. During the trial, anamnesis, laboratory analysis, and physical exams were gathered prospectively. Physical and anthropometric measurements were taken by competent professionals while the individuals were barefoot and dressed in light clothing. Urine and blood samples were taken before and after the procedures, as well as during the follow-up period. Blood samples were taken in the morning (after a 12-h fast). These samples were allowed to remain at room temperature for 30 min before being centrifuged to separate the serum.

An automated analyzer (Cell-Dyn Sapphire and Architect ci16200, Abbott) was used to perform hematimetric and biochemical assays. Insulin was measured using a chemiluminescent immunoassay (Advia Centaur, Siemens). Nephelometry (Immage 8000, Beckman Coulter) was used to measure high sensitive C reactive protein (hs-CRP) and lipoprotein (a) (Lp [a]). All samples were run twice, and the intra- and inter-assay variance coefficients were less than 10%.

Levels of adiponectin, interleukin-6 (IL-6), interleukin-1beta (IL-1beta), and tumor necrosis factor-alpha (TNF-α) were measured by an optimized magnetic bead-based multiplex assay for the Luminex® platform (LXSAHM, Bio-Techne R&D Systems, S.L.U.). All analyses were performed according to the manufacturer’s protocols.

### Estimation of InsP6 consumption

A validated questionnaire (the Food Frequency Questionnaire (FFQ)) was used to measure InsP6 consumption, which took into consideration 10 key sources of InsP6 (whole grains, legumes, and nuts), the serving size of each item, and the InsP6 concentration of each item [23, 24]. Low InsP6 intake was defined as consuming significant sources of InsP6 less than three times per week.

### Measurement of urinary InsPs

Urinary InsPs was determined 2 hours after the first urine of the day using a previously established indirect phytate assay based on the aluminum-pyrocatechol violet (Al-PCV) technique [25]. Because this determination does not distinguish between InsP6 and other InsPs, the observed parameter is referred to as “phytic acid equivalents”.

### Statistical analysis

Data are presented as means (standard deviations), medians (interquartile ranges), or numbers (percentages). Intergroup comparisons at baseline (T0, before intervention) were analyzed using the independent-samples t test or the Mann–Whitney *U* test for continuous variables, and the chi-square test or Fisher’s exact test for categorical variables. Intragroup differences (before [T0] vs. after intervention [T1]; after intervention [T1] vs. after follow-up [T2]) were evaluated using a paired-samples *t* test or Wilcoxon’s signed-rank paired test for continuous variables, and the McNemar test for dichotomized variables. Intergroup comparisons ([T1] and [T2]) were assessed using analysis of covariance and Fisher’s exact test, with adjustment for changes in categorical and continuous variables according to baseline values. Bivariate associations were evaluated by Pearson’s or Spearman’s correlation coefficients. The procedure described by Benjamini and Hochberg was used to correct for multiple comparisons of the 17 variables tested with a false discovery rate of 20% [26, 27]. Statistical analyses were performed using SPSS version 23.0 (SPSS Inc., Chicago, IL, USA).

### Ethical considerations

The Research Committee of Hospital Son Llàtzer and the Balearic Islands Research Ethics Committee [CEI-IB] (IB1933/12 PI) approved the study design. Before participating, all patients provided written informed permission. All tests were carried out in conformity with the applicable standards and regulations. EudraCT no. 2017-003609-16, which was validated on February 22, 2018, is the clinical trial number. U1111-1201-5736 is the Universal Trial Number (UTN). Other earlier clinical study outcomes have previously been published [28].

## Results

### Baseline characteristics of patients

Thirty-nine patients (23 females and 16 males) completed the clinical study (Table 1). The median age was 65 years (interquartile range (IQR): 54–71) and the mean duration of T2DM was 11 years (IQR: 6–14). Six patients (15.8%) consumed alcohol and five patients smoked (13.2%). Considering complications associated with T2DM, eight patients (20.5%) presented at least one complication, with retinopathy (16.2%) being the most prevalent.

**Table 1:** Baseline characteristics of patients (*n* = 39).

Baseline characteristics (*n* = 39)		
Age (years)		65 (54–71)
Duration of DM (years)		11 (6–14)
Sex (female)		23 (60.5%)
DM complications		7 (18.9%)
Type of complications	Erectile dysfunction	1 (2.7%)
	CKD	1 (2.7%)
	Neuropathy	2 (5.4%)
	Retinopathy	6 (16.2%)
Comorbidities	Smoking (ex or yes)	5 (13.2%)
	Alcohol (ex or yes)	6 (15.8%)
	Dyslipidemia	25 (65.8%)
	Hypertension	21 (55.3%)
Medication use	Insulin	19 (59.4%)
	Oral antidiabetic drugs (OAD)	30 (93.8%)
α-GLP-1		7 (21.9%)
Statins		30 (78.9%)
Fibrates		2 (6.3%)
Antihypertensives		21 (55.3%)
Dyslipidemia treatments		23 (71.9%)

Each value is given as median (interquartile range) or percentage (%).

Regarding medication use, thirty patients (93.8%) were on oral antidiabetic agents, and nineteen (59.4%) used insulin therapy. Twenty-one (55.3%) were on antihypertensive therapy, and twenty-three (71.9%) were treated with lipid-lowering agents. During the study period, there were no changes in medication usage (Table 1).

### Effect of dietary InsP6 on patients with T2DM

**Fig. 2:**
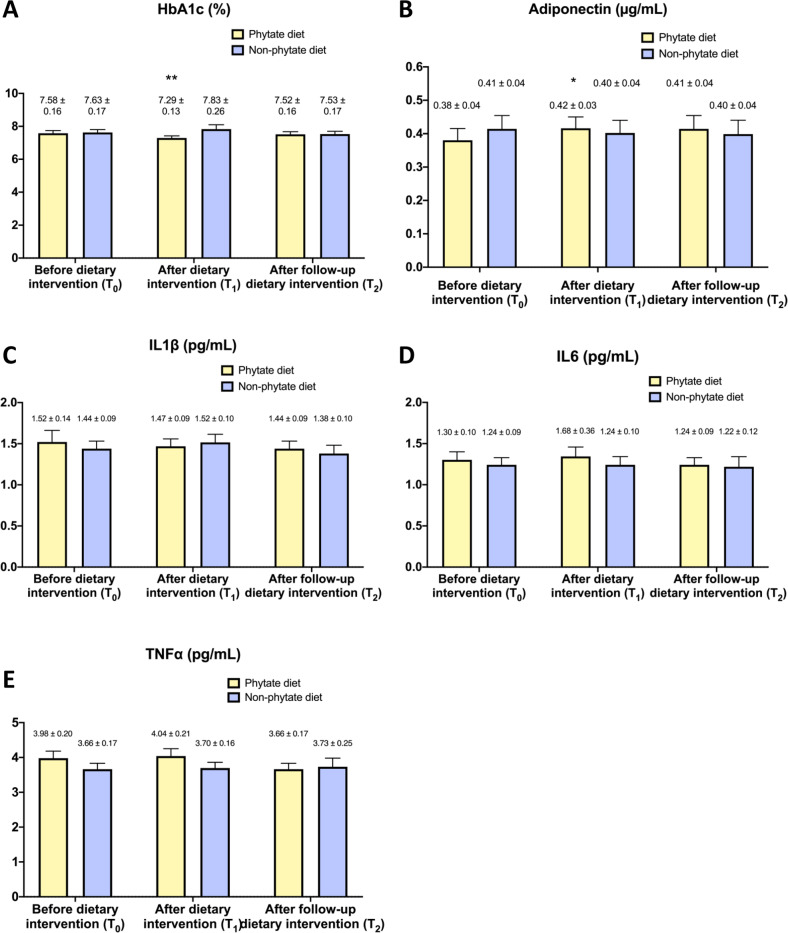
Effect of phytate on HbA1c, Adiponectin, IL1beta, IL6 and TNF-alpha. Effect of phytate diet on serum levels of HbA1c (**A**), Adiponectin (**B**), IL1 (**C**), IL-6 (**D**), and TNF-alpha (**E**). Values are expressed as means and SEM. Intragroup differences (T0 vs. T1, T1 vs. T2) were assessed using a paired-sample Wilcoxon signed-rank test or T-paired test (**P value <0.05 for T1 vs. T0 & T1 vs.T2, *P value <0.05 for T1 vs. T0). Intergroup differences (phytate diet vs. non-phytate diet) were assessed using the Mann–Whitney *U* test or *T* student for the before-dietary intervention group, and analysis of covariance after adjusting for baseline levels for after and follow-up dietary intervention groups (#). Benjamini–Hochberg procedure was used to correct for the false discovery rate.

Table 2 shows anthropometric and laboratory values before starting each diet. As can be seen, no differences were found between groups at baseline (T0, before intervention). Furthermore, the two groups had similar levels of HbA1c, adiponectin, IL-1beta, IL-6, and TNF-alpha (Fig. 2).

**Table 2:** Anthropometric and laboratory values before starting each diet.

	Before phytate diet (T0)	Before non-phytate diet (T0)	Intergroup *P* value
	Median (Q1–Q3)	Median (Q1–Q3)	
Weight (kg)	88 (81–98)	87 (78–95)	0.751
BMI (kg/m^2^)	37 (34–41)	36 (33–40)	0.779
Waist (cm)	106 (102–118)	107 (102–114)	0.733
Hip (cm)	108 (102–115)	110 (103–116)	0.824
Waist/hip	1.00 (0.96–1.01)	0.99 (0.96–1.02)	0.393
Glucose (mg/dL)	175 (140–199)	170 (130–211)	0.686
Total cholesterol (mg/dL)	159 (138–190)	171 (141–203)	0.547
LDL cholesterol (mg/dL)	89 (79–114)	100 (79–124)	0.524
HDL cholesterol (mg/dL)	41 (34–50)	39 (35–45)	0.345
Triglycerides (mg/dL)	117 (97–199)	136 (104–170)	0.663
Insulin (µUI/mL)	19 (11–40)	20 (12–41)	0.864
Lipoprotein A (mg/dL)	21.7 (4.4–82.5)	14.9 (3.9–64.0)	0.363
hs-CRP (mg/dL)	0.36 (0.19–0.76)	0.36 (0.19–0.57)	0.697
Iron (µg/dL)	70 (55–95)	79 (61–102)	0.286
Ferritin (ng/mL)	49 (20–129)	55 (20–95)	0.946
Transferrin (mg/dL)	263 (247–289)	265 (241–284)	0.298
Transferrin saturation index (%)	19 (16–25)	21 (17–27)	0.224
Vitamin B_12_ (pg/mL)	390 (240–505)	333 (269–434)	0.677
Folate (ng/mL)	8.0 (5.4–11.6)	7.1 (4.9–9.2)	0.430
Leukocytes (×10^9^/L)	8.0 (7.2–10.0)	8.4 (6.6–9.3)	0.376
Lymphocytes T1 (×10^9^/L)	2.6 (2.1–3.7)	2.4 (1.9–2.9)	0.286
Lymphocytes (%)	33 (27–37)	30 (29–39)	0.967
Platelets (×10^6^/L)	257 (234–299)	251 (223–300)	0.648
HOMA IR	0.98 (0.47–1.24)	0.88 (0.49–1.60)	0.884

Each value is given as median (interquartile range). The significance of differences between groups (intergroup comparison) was determined using Mann–Whitney *U* test or independent samples *t* test.

After dietary intervention (T1, at 3 months), patients given the InsP6 diet had a significant decrease in serum levels of HbA1c (7.58 ± 0.16% to 7.29 ± 0.13%; *P* < 0.05) and an increase adiponectin levels (0.38 ± 0.04 to 0.42 ± 0.03 µg/mL; *P* < 0.05) whereas no differences were found in IL-1beta, IL-6 and TNF-alpha (Fig. 2). At 6 months (T2, after follow-up period) no significant changes were found in the levels of these proteins from T0 to T2.

Table 3 and Table 4 summarize the changes in the other risk factors after the dietary intervention (T1 vs. T0) and after the follow-up period (T2 vs. T1), respectively. A slight increase in triglycerides was observed in InsP6 group after intervention. After the follow-up period, the triglyceride levels decrease again in InsP6 group. Nevertheless, these changes in the triglyceride levels do not remain statistically significant after adjusting the false discovery rate by Hochberg–Benjamini procedure. No significant changes in the other clinical and biochemical parameters were observed (Tables 3 and 4).

**Table 3:** Changes in vascular risk factor after the diet intervention (T1, 3 months).

	After phytate diet (T1)		After non-phytate diet (T1)		
	Median (Q1–Q3)	T1 vs T0 *P* value	Median (Q1–Q3)	T1 vs T0 *P* value	Intergroup *P* value
Weight (kg)	87 (80–93)	0.572	87 (79–93)	0.457	0.828
BMI (kg/m^2^)	36 (33–39)	0.638	36 (33–39)	0.650	0.666
Waist (cm)	108 (102–116)	0.358	108 (102–115)	0.781	0.371
Hip (cm)	110 (103–116)	0.140	108 (103–118)	0.307	0.195
Waist/hip	1.00 (0.93–1.02)	0.876	0.99 (0.97–1.01)	0.480	0.478
Glucose (mg/dL)	171 (137–207)	0.448	154 (123–203)	0.760	0.548
Total cholesterol (mg/dL)	166 (147–190)	0.950	161 (134–180)	0.073	0.155
LDL cholesterol (mg/dL)	89 (76–117)	0.725	90 (67–115)	0.451	0.380
HDL cholesterol (mg/dL)	40 (34–47)	0.080	42 (36–48)	0.210	0.060
Triglycerides (mg/dL)	161 (88–226)	0.210	122 (97–159)	0.182	0.045
Insulin (µUI/mL)	17 (12–35)	0.396	20 (13–33)	0.811	0.330
Lipoprotein A (mg/dL)	19.2 (3.9–83.8)	0.225	17.3 (4.1–54.6)	0.245	0.225
hs-CRP (mg/dL)	0.30 (0.21–0.73)	0.446	0.36 (0.18–0.70)	0.105	0.830
Iron (µg/dL)	74 (56–97)	0.035	70 (57–91)	0.170	0.194
Ferritin (ng/mL)	46 (25–106)	0.736	45 (18–80)	0.347	0.935
Transferrin (mg/dL)	262 (239–282)	0.214	265 (251–287)	0.377	0.189
Transferrin saturation index (%)	21 (16–26)	0.061	20 (16–25)	0.309	0.169
Vitamin B_12_ (pg/mL)	391 (266–473)	0.219	336 (242–429)	0.399	0.915
Folate (ng/mL)	6.9 (5.1–12.6)	0.723	6.8 (5.3–8.8)	0.899	0.954
Leukocytes (×10^9^/L)	8.5 (6.5–9.5)	0.416	7.8 (6.4–9.6)	0.681	0.372
Lymphocytes T1 (×10^9^/L)	2.5 (1.9–3.2)	0.470	2.4 (1.9–2.8)	0.450	0.295
Lymphocytes (%)	32 (27–36)	0.399	31 (27–37)	0.865	0.456
Platelets (×10^6^/L)	263 (226–322)	0.688	257 (217–319)	0.407	0.358
HOMA IR	0.80 (0.54–1.04)	0.473	0.72 (0.52–1.45)	0.473	0.266

Un-adjusted within groups changes (before, T0 vs. after intervention, T1) are given as median (interquartile range). Intra-groups analysis (T0 vs. T1) used a paired-sample Wilcoxon signed-rank test or paired-samples *t* test to determine the significance of differences. Intergroup analysis (InsP6 diet vs, non-InsP6 diet) used analysis of covariances and comparison between groups after adjusting for baseline levels to determine the significance of differences. After Bonferroni correction, the statistical threshold was adjusted to *P*<0.05/24 = 0.002.

**Table 4:** Changes in vascular risk factor after follow-up period (6 months).

	After follow-up phytate diet (T2)		After follow-up non-phytate diet (T2)		
	Median (Q1–Q3)	T1 vs T2 *P* value	Median (Q1–Q3)	T1 vs T2 *P* value	Intergroup *P* value
Weight (kg)	87 (80–95)	0.369	87 (79–94)	0.212	0.757
BMI (kg/m^2^)	36 (33–40)	0.463	36 (33–39)	0.225	0.844
Waist (cm)	109 (102–114)	0.255	107 (102–115)	0.653	0.465
Hip (cm)	110 (103–116)	0.256	108 (103–117)	0.644	0.274
Waist/hip	0.99 (0.96–1.02)	0.064	0.99 (0.97–1.01)	0.636	0.099
Glucose (mg/dL)	174 (135–211)	0.823	166 (135–186)	0.928	0.820
Total cholesterol (mg/dL)	171 (141–203)	0.074	165 (140–191)	0.707	0.475
LDL cholesterol (mg/dL)	100 (79–124)	0.036	91 (70–115)	0.278	0.189
HDL cholesterol (mg/dL)	38 (35–45)	0.665	44 (38–49)	0.239	0.621
Triglycerides (mg/dL)	136 (104–184)	0.027	124 (100–171)	0.127	0.033
Insulin (µUI/mL)	20 (12–46)	0.357	20 (13–33)	0.177	0.698
Lipoprotein A (mg/dL)	14.9 (3.9–64.0)	0.071	12.5 (2.9–67.4)	0.567	0.467
hs-CRP (mg/dL)	0.33 (0.19–0.57)	0.537	0.42 (0.22–0.69)	0.566	0.054
Iron (µg/dL)	79 (57–102)	0.561	72.00 (61–88)	0.967	0.976
Ferritin (ng/mL)	55 (20–102)	0.405	32 (18–82)	0.027	0.308
Transferrin (mg/dL)	268 (241–287)	0.897	259 (249–276)	0.139	0.337
Transferrin saturation index (%)	22 (17–27)	0.381	20 (17–25)	0.711	0.915
Vitamin B_12_ (pg/mL)	340 (269–470)	0.694	366 (266–460)	0.281	0.627
Folate (ng/mL)	6.7 (4.1–9.7)	0.051	7.2 (6.2–8.4)	0.344	0.041
Leukocytes (×10^9^/L)	8.4 (6.6–9.8)	0.144	8.1 (6.5–9.4)	0.821	0.880
Lymphocytes T1 (×10^9^/L)	2.6 (2.0–3.0)	0.215	2.4 (1.8–3.2)	0.530	0.655
Lymphocytes (%)	31 (28–39)	0.284	32 (27–35)	0.327	0.173
Platelets (×10^6^/L)	259 (224–304)	0.280	270 (242–308)	0.095	0.457
HOMA IR	0.94 (0.50–1.70)	0.153	0.65 (0.49–1.43)	0.208	0.614

Un-adjusted within groups changes (after intervention, T1 vs. after follow-up period, T2) are given as median (interquartile range). Intra-groups analysis (T1 vs. T2) employed a paired-sample Wilcoxon signed-rank test or paired-samples *t* test to determine the significance of differences. Intergroup analysis (InsP6 diet vs. non-InsP6 diet) employed analysis of covariances and comparison between groups after adjusting for dietary intervention levels to determine the significance of differences.

### Effect of dietary InsP6 on urinary excretion of InsPs

**Fig. 3:**
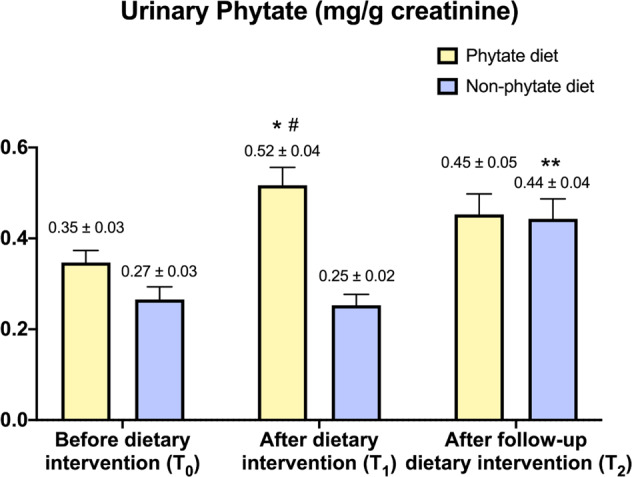
Effect of phytate diet on urinary levels of InsPs. Values are expressed as means and SEM. Intragroup differences (T0 vs. T1, T1 vs. T2) were assessed using a paired-sample Wilcoxon signed-rank test (*P value <0.05 for T0 vs. T1, **P value for T2 vs. T1 & T2 vs.T0). Intergroup differences (phytate diet vs. non-phytate diet) were assessed using Mann–Whitney *U* test for the before-dietary intervention group, and analysis of covariance after adjusting for baseline levels (#). Benjamini–Hochberg procedure was used to correct for the false discovery rate.

Patients in the InsP6 group exhibited a significant increase in urinary InsPs after dietary intervention (T1: 0.52 ± 0.04 mg/g creatinine) compared to baseline levels (T0: 0.35 ± 0.03 mg/g creatinine) (Fig. 3). After washout period, there was a slight decrease in urinary InsPs for patients in InsP6 group (T2: 0.45 ± 0.05 mg/g creatinine). Patients in the non-InsP6 group had no significant changes of urinary InsPs after dietary intervention (T1 vs. T0).

### Safety and adverse events

All patients exhibited good tolerance to the InsP6 tablets. There were no serious adverse events (death, life-threatening events, or events placing a patient in jeopardy or leading to hospital admission) and no dropouts related to InsP6 supplementation. However, one patient on insulin presented had a severe hypoglycemic event while on the non-InsP6 diet.

## Discussion

This is the first study to report the effect of InsP6 supplementation on adiponectin levels in T2DM patients. Our findings are consistent with other rodent experimental investigations. In a recent study, dietary myo-inositol supplementation boosted adiponectin plasma levels in C57BL/6 mice fed a high-fat diet [29].

Other authors discovered that dietary myo-inositol significantly enhanced blood adiponectin levels, but dietary phytic acid somewhat increased them when rats were fed a high sucrose diet containing 1.02% sodium phytate or 0.2% myo-inositol for 12 days [30]. Furthermore, a recent study observed that taking InsP6 as a sugar tablet resulted in lower urine levels of InsPs than taking InsPs through gavage. As a result, when rats were given sugar, InsP6 was less effectively absorbed [16].

In addition, some “in vitro” results confirm these findings and show that the impact of legumes on insulin sensitivity may be mediated, at least in part, by InsP6 or InsPs. A recent study focused on the nutritional and physiological similarities between InsP6 and myo-inositol, and it showed that myo-inositol and myo-inositol phosphates enhance insulin sensitivity in “in vitro” models with adipocytes by boosting lipid storage capacity, enhancing glucose absorption, and suppressing lipolysis [31]. Surprisingly, this has the same impact as thiazolidinediones, which increase white adipose tissue and make people more insulin sensitive [32]. Several studies [33, 34] have been reported that support the notion that insulin resistance is caused by an inability to handle extra energy by either storing it or burning it for energy.

Accordingly, Inositol, or myo-inositol, and its analog compounds (including InsP6 and the other InsPs) are known to have favorable biological qualities and are now being explored extensively. The impact of these chemicals on diabetic indicators is important, especially given the enormous worldwide cost of treating diabetes mellitus and its consequences. Type 2 diabetes is a chronic condition that is recognized to be characterized by low-grade inflammation, which plays an important role in vascular diabetes complications and development [35, 36]. Changes in this inflammatory state can be identified via biomarkers of inflammation including hs-CRP [37], TNF-alpha [38], IL-6 [39], as well as anti-inflammatory biomarkers such as the adipocyte adiponectin [40].

Myo-inositol hexaphosphate (phytate, InsP6) is a natural chemical found in seeds as a calcium-magnesium salt (phytin) and has been shown to regulate enzymes involved in lipid and carbohydrate metabolism. Furthermore, InsP6 lowers food intake, calorie consumption, and modifies adipose tissue cytokine production [41], potentially decreasing the incidence or preventing diabetic complications. InsP6 supplementation reduces arterial damage by diminishing red cell distribution width, and serum HDL levels increase while blood triglycerides decrease [42]. A recent cross-sectional study found that taking IP6 on a daily basis reduces circulation levels of advanced glycation end products by preventing protein glycation in T2DM patients [28].

We discovered that the InsP6 diet had an effect on blood lipid levels. Depending on the InsP6 salt utilized, different studies have observed varying effects on lipid metabolism. On the one hand, sodium InsP6 inhibited lipase activity, resulting in a considerable drop in total cholesterol and low-density lipoprotein while increasing high-density lipoprotein [43, 44]. Sodium InsP6 salt also lowers cholesterol and triglyceride levels in the liver [43, 44]. However, recent research suggests that the calcium-InsP6 complex does not bind bile acids, which might diminish fecal bile excretion while increasing cholesterol availability [44]. It should be mentioned that we employed naturally occurring calcium-magnesium InsP6. It would be interesting to look into the therapeutic implications of sodium phytate as a dietary supplement further.

InsP6 is linked to inflammation and insulin resistance [20–22, 45]. However, we discovered that the InsP6 diet had no effect on IL-6, IL1-beta, TNF-alpha, hs-CRP, leukocyte count, or insulin resistance. In this regard, earlier research has found a decrease in these inflammatory markers following a diet high in InsP6. As a result, we cannot rule out the possibility that InsP6 has an influence on additional modulators or molecular surrogates of inflammation. It is important to remark that a recent clinical trial in individuals with obesity found that legume supplementation increased plasma adiponectin [13]. This variation was linked to a reduction in urinary 8-Epi-PGF 2 alpha (a secondary end product of peroxidation), but not to alterations in hs-PCR were observed [46].

There were some limitations to the current investigation. The first is the small sample, we only examined 39 patients from a single medical center. Consequently, our findings may be restricted in their generalizability. Another weakness of this study is that the experiment (dietary intervention) was not blinded. As a result, a large multicenter, blinded placebo-controlled trial is required to validate our findings.

**Fig. 4:**
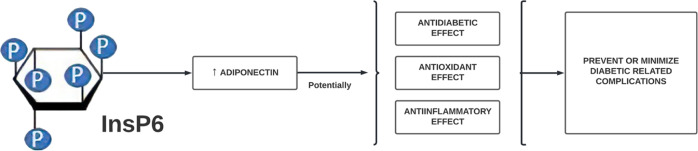
InsP6 supplementation increase adiponectin levels in patients living with T2DM. Thus, could potentially improve glycemic control, decrease inflammation and reduce oxidation potentially reducing or minimizing diabetic-related complications.

As conclusion, our study has demonstrated that daily phytate intake increases adiponectin levels among patients with diabetes type 2. So, our findings indicate that following a phytate-rich diet has beneficial effects on adiponectin concentrations. Adiponectin is an antidiabetic, anti-inflammatory factor and reduces oxidative stress [47, 48]. In addition, adiponectin improves insulin resistance, hyperglycemia, and hyperlipidemia [49]. We hypothesized that inositol and InsP6 supplementation might promote insulin sensitivity by increasing adiponectin levels in the blood. Therefore, we suggest that a diet rich in InsP6 could help to prevent or minimize diabetic-related complications (Fig. 4). Interestingly, the dose of phytate consumed during the InsP6 intervention as pills (3 pills of 380 mg phytate every day) corresponds to the phytate intake in diets rich in legumes and nuts, such as the Mediterranean Diet (1–2 g/day) [24]. Nevertheless, large, long-term, and randomized prospective clinical studies are needed to fully elucidate the benefits and risks of InsP6 consumption in patients with TDM2. Also, mechanistic studies investigating how InsP6 modifies adiponectin concentrations should be warranted.

## Data Availability

The datasets generated and analyzed during the current study are available from the corresponding authors upon reasonable request.
